# Nonlinear multispectral imaging for tumor delineation

**DOI:** 10.1117/1.JBO.25.9.096001

**Published:** 2020-09-03

**Authors:** Elena Beletkaia, Behdad Dashtbozorg, Rubin G. Jansen, Theo J. M. Ruers, Herman L. Offerhaus

**Affiliations:** a Netherlands Cancer Institute, Department of Surgery, Amsterdam, Netherlands; b University of Twente, Faculty of Science and Technology, Enschede, Netherlands

**Keywords:** hyperspectral, classification, tissue characterization, Raman, fluorescence

## Abstract

**Significance:** In breast-preserving tumor surgery, the inspection of the excised tissue boundaries for tumor residue is too slow to provide feedback during the surgery. The discovery of positive margins requires a new surgery which is difficult and associated with low success. If the re-excision could be done immediately this is believed to improve the success rate considerably.

**Aim:** Our aim is for a fast microscopic analysis that can be done directly on the excised tissue in or near the operating theatre.

**Approach:** We demonstrate the combination of three nonlinear imaging techniques at selected wavelengths to delineate tumor boundaries. We use hyperspectral coherent anti-Stokes Raman scattering (CARS), second harmonic generation (SHG), and two-photon excited fluorescence (TPF) on excised patient tissue.

**Results:** We show the discriminatory power of each of the signals and demonstrate a sensitivity of 0.87 and a specificity of 0.95 using four CARS wavelengths in combination with SHG and TPF. We verify that the information is independent of sample treatment.

**Conclusions:** Nonlinear multispectral imaging can be used to accurately determine tumor boundaries. This demonstration using microscopy in the epi-direction directly on thick tissue slices brings this technology one step closer to clinical implementation.

## 1 Introduction

Breast cancer is the second most common cancer accounting for 2.09 million cases and 627,000 deaths worldwide, which is ∼15% of all cancer-related deaths among women in 2018.^1^ Screening programs have led to an increase in the early stage and nonpalpable tumor detection, which increased the number of patients eligible for breast conservative surgery (BCS). BCS aims to remove the tumor while conserving as much healthy breast tissue as possible. BCS is considered successful when all tumor tissue is removed and resection margins of the specimen are tumor negative. However, globally in 10% to 30% of the BCS procedures, tumor positive resection margins are found requiring a secondary surgical procedure to allow re-excision of the suspected areas, as such patients are at risk for local tumor recurrence.^2^ Therefore, one of the key challenges of BCS is to ensure complete removal of the tumor with negative margins. Ensuring clean resection margins avoids patients returning to the operating room (OR) after surgery, reduces patients’ stress, increases hospital’s productivity due to OR availability for new operations, and helps the healthcare system as a whole, by reducing medical costs.

To ensure clear margins preoperative imaging such as mammography or MRI is used to guide the surgeon during BCS, but they are often not sufficient to guarantee radical tumor resection. After surgery, the ultimate margin status is determined by histopathological analysis. Results of this investigation become available several days after the surgical procedure, which means that in case of tumor positive resection margins additional treatment is necessary by either reoperation or additional boost radiotherapy.^3^^–^^6^ There is a clear need for a development of a new tool for direct evaluation of surgical resection margins during the surgery. In case of a tumor positive resection margin, such tool allows immediate surgical intervention during the initial surgical procedure, preventing additional treatments afterward.

Several techniques have been adopted and are currently evaluated as candidates for intraoperative assistance. They include both *in vivo* and *ex vivo* applications and rely on various physical principles to provide information about the makeup of the tissue. Hyperspectral imaging has been successfully used in margin assessment for various types of cancer^7^^,^^8^ and more recently also on breast cancer.^9^ Although hyperspectral imaging is able to image large sections of tissue rather quickly, it suffers from a relatively low spatial resolution. This makes it difficult to detect small tumors and (pre)-cancerous ducts, limiting its accuracy, especially in the case of ductaal carcinoma *in situ*. Other techniques include optical coherence tomography, diffuse reflectance spectroscopy, infrared microscopy, however, most of them can provide limited biochemical information, while lacking temporal and/or spatial resolution.

Another approach is spontaneous Raman scattering, used both *in vivo* through fiber optics^10^ and *ex vivo* on frozen samples.^11^ When applied to the fingerprint spectral region (400 to $2000\;{\mathrm{cm}}^{-1}$), Raman spectroscopy can provide detailed information about the molecular composition, molecular structures, etc.;^12^^,^^13^ the spectra allow for tissue characterization without prior assumptions about its composition. The power of Raman spectroscopy has been demonstrated in cancers of various organs including the kidney,^14^ prostate,^15^ brain,^16^ skin,^17^ and many others as well as diagnosis of cervical dysplasia.^18^ Also multiple studies point out a promising potential of Raman spectroscopy in diagnosis, detection, and classification of breast cancer.^19^^–^^26^ The findings show the possibility not only to discriminate cancerous from healthy tissue, but also to classify the different types of breast cancer and even to grade of the disease.^23^ However, Raman microspectroscopy is limited by long integration times.^27^ Spectral collection times are typically in the range of 0.2 to 30 s.^12^ Raster-scanning methods, used to build Raman spectral images, can take more than $5\;{h/\mathrm{mm}}^{2}$, which is a serious limitation for use in large-area, high-resolution imaging that is critical for clinical practice.

An interesting alternative is surface-enhanced Raman scattering (SERS).^19^ SERS utilizes resonant nanostructures such as gold nanorods that can enhance the local electric field up to $10^{6}$ times, increasing spectral resolution, and decreasing the time needed to collect the spectra. The resonant nanostructures generally have to be administered to the patient in the form of small gold rods, meaning it is not strictly label-free adding another level of complexity when transferred into clinical practice. The gold particles also have a tendency to cluster together, leading to signal inhomogeneity. This can potentially compromise its accuracy and make interpretation of experimental data more difficult.

Nonlinear optical imaging has shown considerable promise in providing intraoperative margin assessment, due to its rapid and label-free detection capabilities.^27^ This includes second harmonic generation (SHG),^28^^–^^30^ two-photon excitation fluorescence (TPF),^31^^,^^32^ and nonlinear Raman spectroscopy methods such as stimulated Raman scattering^33^ and coherent anti-Stokes Raman scattering (CARS).^34^ In comparison to spontaneous Raman scattering, CARS offers a significant signal enhancement, natural confocality, and no signal overlap with one-photon excited fluorescence facilitating fast imaging of relatively large areas of the sample, while still providing detailed information about biochemical composition of the sample. At the same time, the nonlinear nature of CARS permits imaging with subcellular resolution. Moreover, the use of tunable laser enables selection of individual wavenumbers of interest further decreasing data acquisition time.

CARS is primarily used in the high-wavenumber region to detect high densities of ${\mathrm{CH}}_{2}$ groups in biological tissues^13^^,^^33^ because of their large scattering cross sections and high local concentrations in cellular lipids droplets, adipocytes, nervous tissues, breast tissue, fatty liver tissue, and other lipid-related pathologies. In many Raman studies, e.g., Ref. 11, of breast tissue, it was shown that there are spectral differences between normal and breast tissue associated with lipid and protein content: pathological tissues exhibit the presence of more proteins and fewer lipids. The protein concentration can also be tackled using CARS at a selected wavenumber.

Various combinations of nonlinear imaging techniques have been applied to the problem of breast cancer delineation.^17^^,^^35^^–^^43^ Most commonly it involves trying to create histology-like images—“Optical-H&E” or “Pseudo-H&E”—usually through information gathered by SHG, TPF, and/or CARS.^20^^–^^22^ These images replicate the traditional H&E image, providing physicians with information in a way that they are used to and can easily interpret. Human interpretation is still required, but computer-aided diagnostics are being developed to help in this process.^23^^–^^26^^,^^44^^–^^47^

In this study, we aim to validate if nonlinear imaging, mainly CARS imaging in the high-wavenumber region, provides sufficient information to discriminate healthy breast tissue from cancerous and what (combination of) wavenumbers has the most significant discriminative power. As it was shown that spectra of bulk versus sliced tissue are rather different, we used tissue biopsies for all the measurements. An H&E section was used for every sample imaged by CARS and scored by a trained pathologist to provide ultimate tissue classification as the gold standard. Since in the previous studies it was demonstrated that label-free imaging methods and histopathological H&E staining of the same slides showed strong morphological agreement,^34^ we used a multimode—SHG/TFP/CARS—imaging for matching of the CARS spectra with the corresponding H&E tissue type. We tested 17 biopsies from 14 patients. Our results show that CARS spectra of fat cells can be successfully distinguished from connective tissue (collagen fibers and elastin) and other cells with excellent selectivity. However, healthy tissue cells and cells in the cancerous regions show less pronounced differences. Feature importance analysis shows that we can distinguish between cancerous and healthy tissue. First, the CARS is dominant in separating the fat cells, from connective tissue and cells. The SHG is the dominant factor to separate the connective tissue (healthy and cancerous) from the tissue cells. The TPFE with the CARS distinguishes the cancerous cells from the healthy cells. By measuring in the epi-direction on thick tissue slices, we bring this technology one step closer to clinical implementation.

## 2 Materials and Methods

### 2.1 Sample Preparation

The samples were acquired as 5-mm biopsies during routine pathological preparation of the specimen directly after the surgery. The biopsies were snap frozen in liquid nitrogen and subsequently stored at −80°C. A 5-μm frozen slice was used for H&E preparation according to the standard protocol and the rest of the tissue was used for imaging. Tissue samples were thawed at room temperature and placed in a FluoroDish FD35 Petri dish filled with formalin in such a manner that the imaging was conducted on the consecutive slide to the cut for H&E surface enabling matching of the H&E image with the CARS and/or other measurements. The sample was fixated at the bottom of the Petri dish by putting a transparent block of PDMS on top of the sample.

### 2.2 Experimental Setup

The setup consists of a high-power aeroPULSE fiber laser (NKT Photonics)—a passively mode locked fiber laser with a center wavelength of 1032 nm. It has a pulse length of 6 ps and a repetition rate of 80 MHz, with a nominal power output of 10 W. Part of the 1032 power is used as the Stokes beam in the CARS process, whereas the rest is frequency doubled to 516 nm. This light is then used to pump the Levante optical parametric oscillator (OPO) (APE, Germany), which converts part of it into a signal and an idler beam ($\omega_{\text{pump}}=\omega_{\text{signal}}+\omega_{\text{idler}}$). The idler is not used in this setup and is discarded in a beam dump. The signal beam is used as the pump in the CARS process, and hereafter is referred to as “pump beam.” The wavelength of the pump beam can be tuned from 690 to 990 nm and used in the range of 775 to 807 nm to generate CARS range of 2700 to $3200\;{\mathrm{cm}}^{-1}$. A small part of this pump beam is split off and directed into a power meter, which is used to monitor the average output power. These data are used later to correct for power fluctuations during the spectral measurements. Another part of the pump beam is split off and coupled into a spectrometer (Ocean Optics HR2000, the Netherlands), which is used to verify that the desired wavelength is generated. If this is not the case, various parameters on the OPO can be adjusted, such as cavity length, Lyot filter position, and crystal temperature. The Stokes beam can be modulated with an acousto-optic modulator (Crystal Technology 3080-197, USA), and is spatially and temporally overlapped with the pump beam through the use of a delay stage and an appropriate dichroic mirror. The overlapped beams proceed into an Olympus FV300 laser scanning microscope which consists of a scanning box with galvo mirrors, and an Olympus IX71 inverted microscope. The microscope is equipped with a filter wheel, with different combinations of filters and dichroic mirrors. Furthermore, a set of objectives (Olympus 60X/1.2NA water immersion and Olympus 20X/0.75NA air objective) are installed allowing to create an image of $235.7\times 235.7\;\mu m^{2}/\text{frame}$ or $707\times 707\;\mu m^{2}/\text{frame}$, respectively.

For signal detection, a photo multiplier tube (PMT)—Hamamatsu R3896—is placed in the epi-direction. An optional extra (narrowband) filter can be placed directly in front of the PMT (in addition to the filter in the filter wheel) to reject background light from the SHG signal.

### 2.3 Homodyne Detection

The TPFE signal partially overlaps spectrally with the CARS signal. The majority of the TPFE can be collected using a spectral filter but the minor overlapping part presents a substantial background to the smaller CARS signal. To separate the contribution of the TPFE from the CARS signal, homodyne detection is used, in which the Stokes beam in the CARS process is modulated (on/off). As a result, the CARS signal, which depends on the presence of the Stokes, is modulated as well whereas the TPFE that is generated predominantly by the pump beam remains constant. By detecting only the modulated part of the signal around the CARS wavelength, we separate the CARS portion. We separately verified that the Stokes beam alone does not generate any significant TPFE. The Stokes beam is modulated by an acoustic optical modulator, with a square wave at 1 MHz generated by an SFG-2110 function generator (GW Instek, Taiwan). The resulting signal from the PMT is demodulated on a HF2LI Lock-in amplifier (Zurich Instruments, Switzerland). A low-pass filter at roughly the same frequency as the pixel sampling frequency is applied, to average the demodulated signal over a single pixel.

### 2.4 Data Acquisition

An overview of SHG, TPF, or CARS (2850 or 2940) image acquired with the 60× objective was recovered by stitching the individual frames. Measured with 256×256  pixels resolution these images feature a spatial resolution of 0.91  μm. For the hyperspectral scan, a single frame was measure repeatedly, while changing the CARS wavelength over the range of 2700 to $3200\;{\mathrm{cm}}^{-1}$. A combination of a stitching scan and hyperspectral scan (hyperstitch) was made on selected regions of interest.

To accurately reconstruct the Raman spectrum, the measured intensity was calibrated with respect to the output power of the OPO and Stokes beam power, thus obtaining a spectrum that is independent of input power. The data were also corrected for the constant offset on the ADC, of about 50 mV and variation of the PMT voltages were used for signal detection. The stitched images were corrected for the uneven illumination (bright in the center and less bright at the edges) by fitting a fourth order 2-D polynomial to the overall averaged intensity of all frames and dividing the individual frames by this shape.

#### 2.4.1 Support vector machine recursive feature elimination

Precision and speed tend to be conflicting requirements so that it is important to know which type of signal or which wavelengths influence the classification the strongest and which measurements might be redundant. Many strategies have been developed for this feature selection and have been implemented as MATLAB routines,^48^^,^^49^ support vector machine recursive feature elimination (SVM-RFE) is one such method.^50^ It is designed to distinguish only binary problems (whereas we classify into four tissue types) but can be extended by adding the weights of the binary subclassification to arrive at an overall classification.

#### 2.4.2 Feature importance analysis

When the number of features (intensity at different wavenumbers) is very large relative to the number of samples in the dataset, some classifiers struggle to train effective models. Feature extraction and feature selection techniques can be used to avoid the curse of dimensionality. Feature extraction creates a new, smaller set of features that still captures most of the useful information, while feature selection keeps a subset of the original features. The scheme used in this paper is the sequential forward selection (SFS) method.^50^^–^^52^ This method starts with an empty feature set and, for each step, the best feature that satisfies a criterion function is included with the current feature set. The accuracy of an SVM classifier is taken as the criterion function. The SFS algorithm stops when the number of included features reached the predefined maximum number of features (set to 30 for this work).

Since the SFS algorithm may result in local decisions, the feature selection is repeated for 20 times to obtain a globally optimal solution. The final feature set is obtained by selecting the wavenumbers with the highest occurrence number after 20 iterations.

## 3 Results

### 3.1 Tissue Fixation

**Fig. 1:**
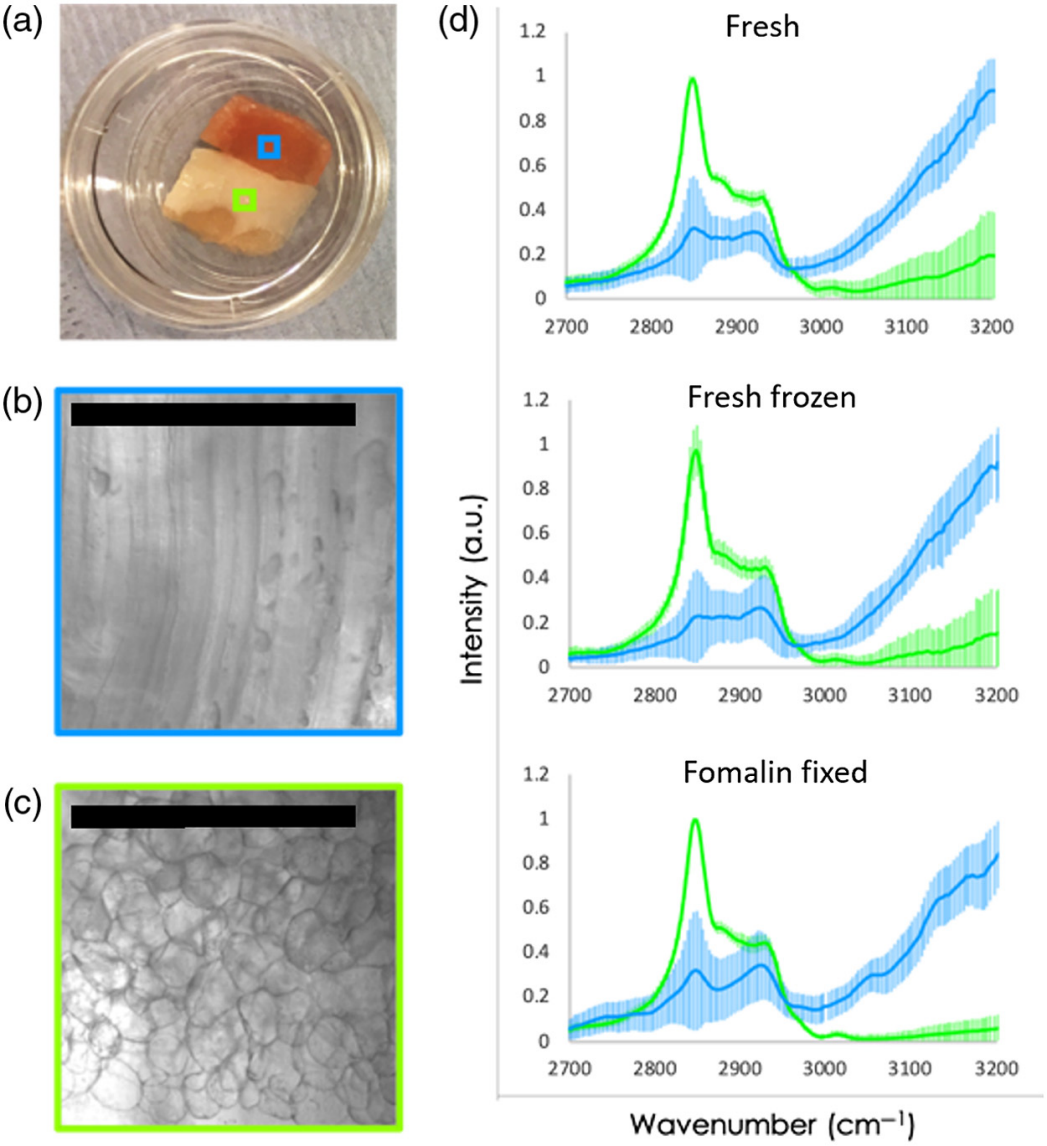
Spectra for different fixation methods: (a) sample with meat (above) and fat (below), (b) enlarged section of meat, scalebar is 500  μm, (c) enlarged section of fat, scalebar is 500  μm, and (d) CARS spectra for different fixation methods on meat (blue) and fat (green). The error bars indicate standard deviation over the measured region.

**Fig. 2:**
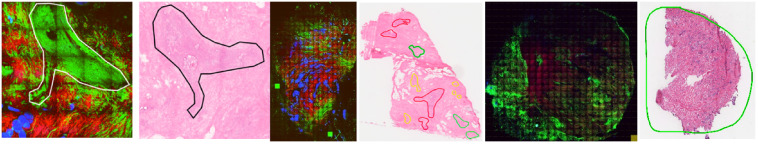
Three examples of matching consecutive tissue slices. The images in the left of each pair are measured with CARS and in the right are stained with HE. The false color in the left uses blue for CARS at $2850\;{\mathrm{cm}}^{-1}$, red for SHG, and green for TPF. The circled areas are matched. In the first pair of images, the circled area contains healthy cells. In the middle pair of images, the areas circled in red indicated suspicious cells (unpon further inspection labelled as healthy), yellow are healthy cells, and the green areas indicate inflamed cells. The image in the right is all designated as tumor cells.

In different studies, it was shown that formalin, as well as liquid nitrogen fixation, can generate some additional peaks in the fingerprint region while altering the intensity of existing peaks which would make the data analysis and results translation to the nonfixed tissue more complex. To examine whether a similar effect happens in the high-wavenumber region, we did measurements using a model tissue—pork. We used pork as fresh, formalin-fixed, and fresh frozen samples imitating regular pathology tissue preparation. For all samples, a CARS spectrum was acquired in a high-wavenumber region 2700 to $3200\;{\mathrm{cm}}^{-1}$ (see Fig. 1). Our results show that there is no significant difference in spectra acquired from samples treated in different ways. Moreover, our measurements indicated a clear differentiation of different types of pork tissue, namely fat and meat. From Fig. 1(c), it is clear that fat tissue has a dominant peak at $2850\;{\mathrm{cm}}^{-1}$, corresponding to ${\mathrm{CH}}_{2}$ stretch, while in the meat tissue Fig. 1(b), the signal from OH stretch (water) is more pronounced. Furthermore, we tested a combined effect of the fixation methods: liquid nitrogen fixed tissue was thawed at room temperature and then fixed in formalin. When CARS spectra were acquired from these samples, no difference was observed when compared to fresh or formalin or liquid nitrogen fixed samples. These results enabled us to develop a protocol for patient tissue handling and use that is independent from the surgery planning. This protocol was implemented as described in M&M. In short, a 5-mm biopsy was acquired directly after surgery and snap frozen in liquid nitrogen. A frozen section H&E was prepared and the consecutive slice was used for the CARS measurements at room temperature in formalin. Such imaging of the consecutive planes enabled matching of the H&E and CARS images (see Fig. 2).

### 3.2 Tissue Spectra

**Fig. 3:**
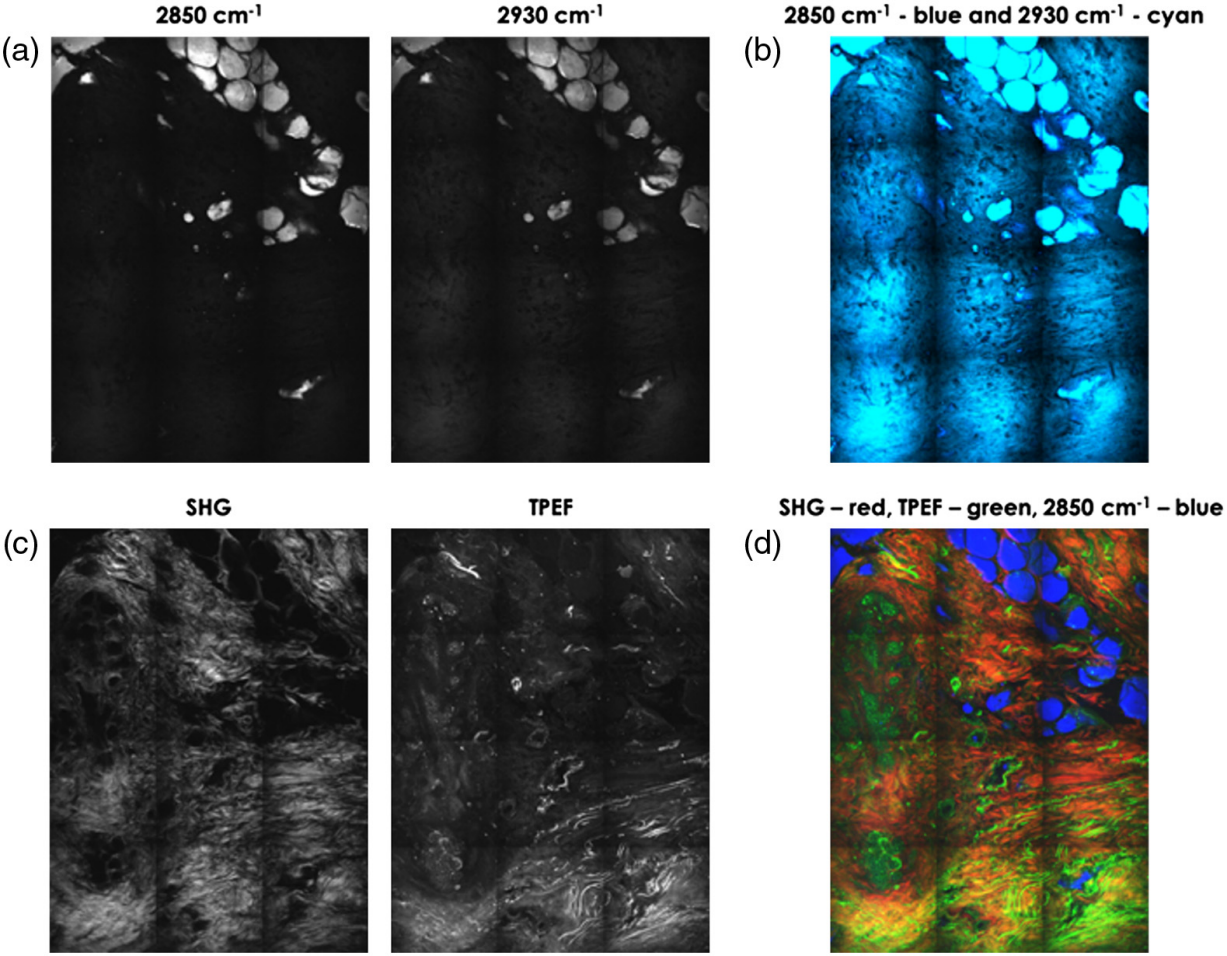
Different signals from the same region: (a) CARS signal at 2980 and $2930\;{\mathrm{cm}}^{-1}$, respectively, (b) these two overlaid in blue and cyan, respectively, (c) the SHG and TPF signal, and (d) three signals overlaid where SHG is shown in red and TPF in green and the CARS at $2850\;{\mathrm{cm}}^{-1}$ is shown in blue. The squares are caused by stitching different images together.

**Fig. 4:**
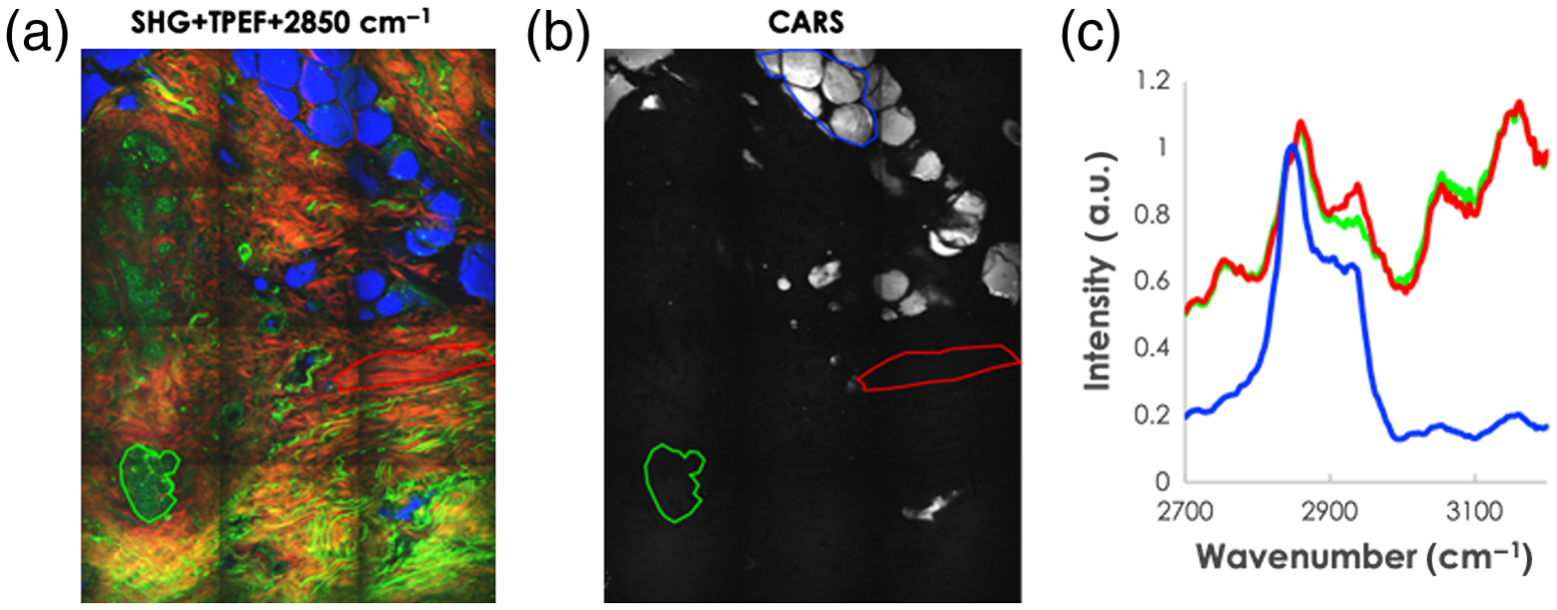
CARS spectra for three tissue types: (a) the combined image, (b) the CARS image at $2850\;{\mathrm{cm}}^{-1}$, and (c) the average spectra for the blue (fat), green (connective tissue), and red (cells) regions.

Matching the H&E slides to the microscopic/spectroscopic measurements is not a trivial challenge. Securing consecutiveness of the observation planes and orientation was a great aid; however, tissue deformation could not be eliminated. We created stitched images of the samples using CARS at $2850\;{\mathrm{cm}}^{-1}$ to highlight the lipids, SHG to highlight the connective tissue, and TPF to highlight cells and connective tissue (i.e., elastin). The composite of these images (a representative example is shown in Fig. 3) was used to find a morphological match between the sample and its corresponding H&E. Due to major tissue deformation, it appeared impossible to make a full sample match; however, the regions of interest for further examination could be selected. For each selected region of interest (ROI), CARS measurements were performed from 2700 to $3200\;{\mathrm{cm}}^{-1}$. In these data sets, three main tissue groups were defined: fat, connective tissue, and cells; and spectra for each group were reconstructed (see Fig. 4).

Notably, stitched images exhibit uneven illumination, which complicates the data analysis. Several elaborate methods have been proposed over the years to compensate for this nonuniform illumination problem postacquisition.^46^^,^^47^ However, these are relatively complicated and difficult to implement. In this study, we first subtracted the constant background on the PMT. Then the average intensity distribution over all the individual frames of the images was calculated and smoothed by fitting a fourth-order two-dimensional polynomial to it. The frames were corrected by dividing them by this average intensity distribution, which yields the results shown in Fig. 4. This correction was applied to all collected measurements. Although it might seem more appropriate to measure a homogenous sample and use that as a reference for the intensity distribution, we found that the distribution showed day to day variation. We tentatively assign these to variation in alignment and differences in imaging depth for different samples. The averaging procedure that we use takes no extra time and reduces the stitching errors to an acceptable level.

**Fig. 5:**
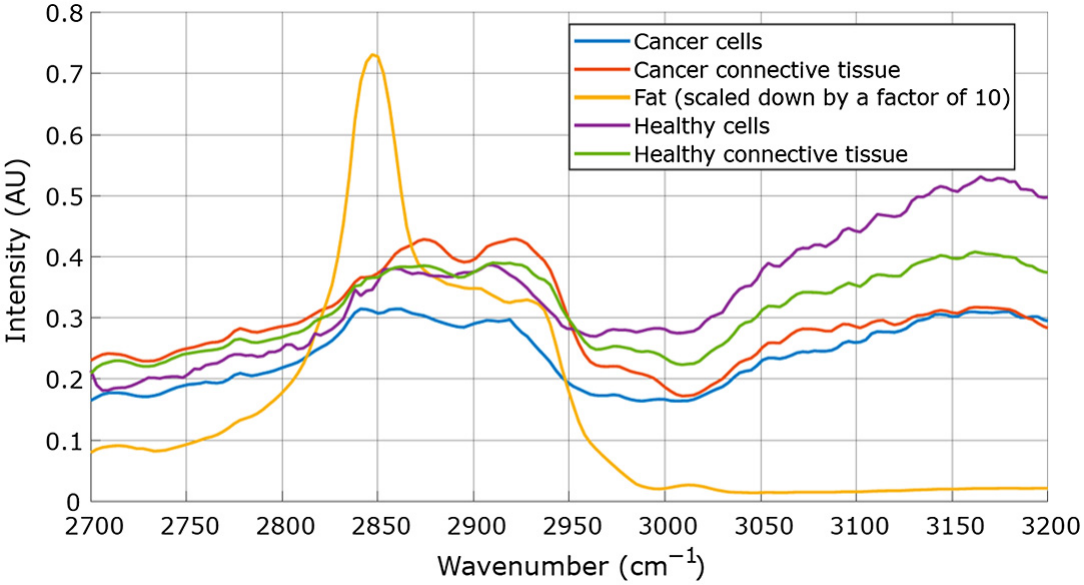
Averaged CARS spectra for different regions of interest where the annotation comes from the matched pathology.

In total, 16 samples from 14 patients were examined of which 4 were biopsies acquired from the healthy tissue and 12 samples were acquired from cancerous tissue. In total, 396,646 spectra were collected and split into five distinct classes (fat, cancer cells, cancer connective tissue, healthy cells, and health connective tissue). To reduce noise, a block of 4×4  pixels (an area of 3.7×3.7  μm) was averaged to produce one spectrum. Figure 5 presents the averaged CARS spectra collected from all the tissue samples, averaged over areas that were assigned by pathology on the adjoining slice as indicated in Figs. 2 and 4. From these spectra, it is clear that the fat tissue has the most distinct profile. As expected, the $2850\;{\mathrm{cm}}^{-1}$ peak is most pronounced making this spectrum easily distinguishable from the spectra of other tissue types. When evaluating spectra from cells and connective tissue, the differences are much less pronounced, however, there is a difference in cells’ spectra collected from healthy-tissue biopsy versus cancerous tissue biopsy (see Fig. 5 blue versus purple line). The same is true when comparing the spectra collected from healthy connective tissue versus connective tissue in cancerous tissue. In accordance with the results previously reported in the literature,^39^^,^^41^ the water/fat ratio (signal at $3150\;{\mathrm{cm}}^{-1}/\text{signal}$ at $2845\;{\mathrm{cm}}^{-1}$) in the cancerous tissue is lower than in the healthy tissue.

**Fig. 6:**
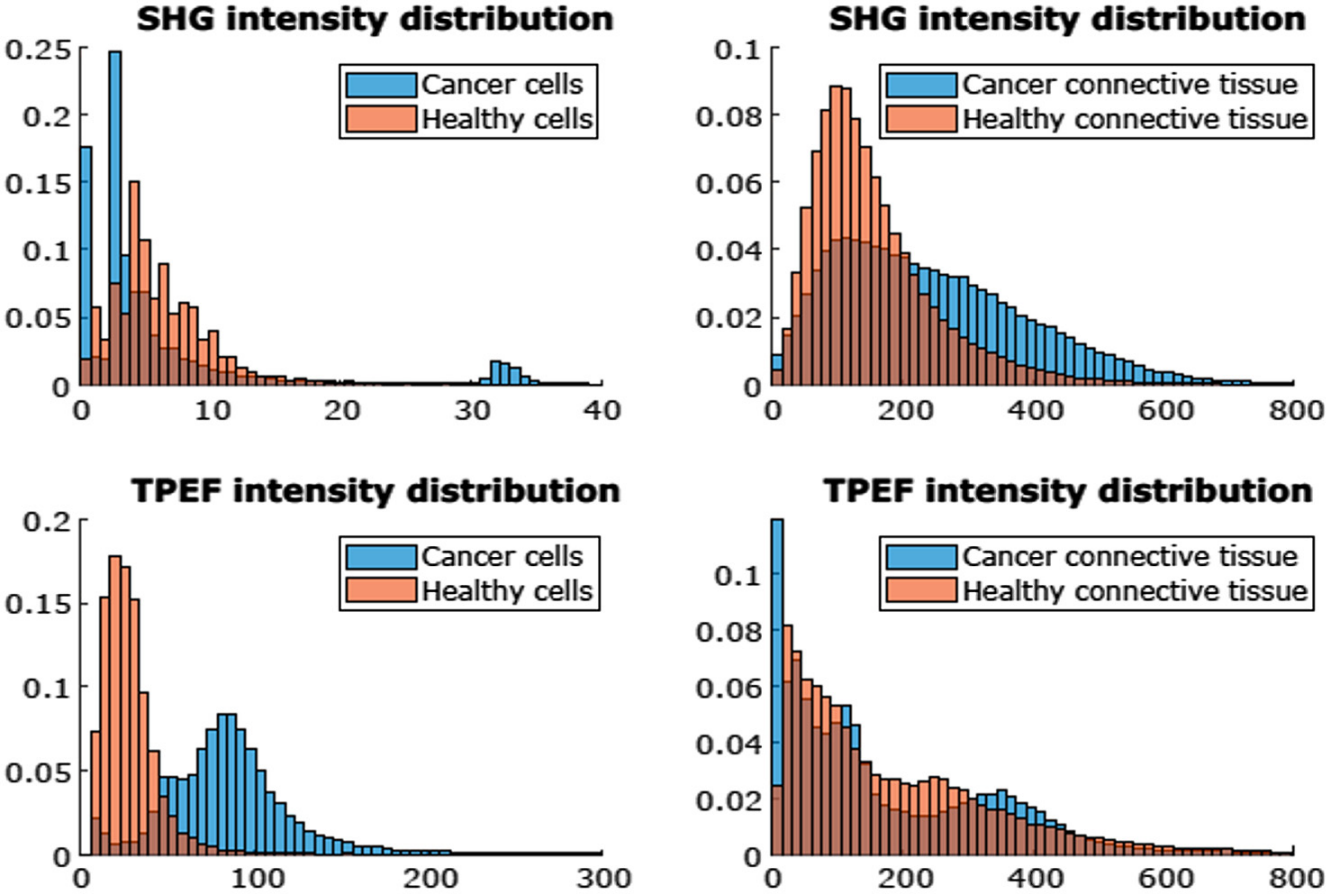
Histograms showing the relative frequency of the SHG and TPF intensities (digitized PMT signals, where 1 V=4096) for the different tissue classes. The horizontal axis is consistent so that the SHG and TPFE signal levels are on the same scale and can be compared. Fat is not included as it does not produce SHG or TPF.

Additionally, the intensity distribution of the SHG and TPF signals was examined (see Fig. 6). As expected, the SHG signal for cells has lower intensity than for connective tissue and there is no distinct difference in SHG distribution between cancerous and healthy cells. In the case of connective tissue, there is more signal of lower intensity for the healthy tissue, while cancerous tissue exhibits a more even distribution. For the TPF signal, the connective tissue does not show much difference between cancerous and healthy tissue. In the case of cells, the healthy cells have a peak in the low-intensity region while the cancer cells have a more pronounced peak at the high-intensity region. Despite these distribution differences, the SHG and TPF signals alone do not contain sufficient discriminative power for tissue classification.

### 3.3 Machine Learning Analysis

For a more quantitative analysis, we addressed a machine learning analysis. In this study, we used two methods: (1) recursive feature elimination (RFE)^50^ and (2) feature importance analysis.^51^^,^^52^ In both cases, we aimed to identify the wavenumbers with the highest discriminative power as well as the minimum number of wavenumbers required for the best result.

#### 3.3.1 SVM-RFE

Due to the limited number of healthy tissue samples that were measured during this research, there are comparatively few spectra of healthy cells and fat. Thus for fat and healthy cells, SVMs were trained with all available spectra. For the other tissue classes, a random subset of 30,000 spectra for each tissue class was taken for the training of the SVMs.

The SVM-RFE feature selection algorithm repeatedly builds an SVM model to classify the input data and rejects the features with the lowest weights. Following this algorithm, 10 most important wavenumbers were identified (2943, 2892, 3192, 3093, 2994, 2700, 2829, 3042, 2748, and $3144\;{\mathrm{cm}}^{-1}$). To avoid adjacent wavenumbers, a minimum spectral distance of 40 inverse centimeters was required, which corresponds to the width of the Raman peaks in this region.

As an SVM is inherently a binary classifier, a single SVM can only be trained to separate two classes. For more than two classes, the problem has to be broken down into cascading binary problems. Each binary problem can then be solved by a single SVM, and a majority vote of all the SVMs decides the final classification. For training purposes, SHG and TPF data were also included for some of the tissue classes resulting in a total number of 10 SVM classifiers (Table 1).

**Table 1:** The 10 SVMs used in the classification scheme, along with their respective input data. Not all available data are given to all SVMs, as this proved detrimental to the scheme’s performance.

SVM	Input class 1	Input class 2	Input data
1	Cancer cells	Cancer connective tissue	SHG + CARS
2	Cancer cells	Fat	TPF + CARS
3	Cancer cells	Healthy cells	TPF + CARS
4	Cancer cells	Healthy connective tissue	SHG + CARS
5	Cancer connective tissue	Fat	SHG + CARS
6	Cancer connective tissue	Healthy cells	SHG + CARS
7	Cancer connective tissue	Healthy connective tissue	SHG + CARS
8	Fat	Healthy cells	TPF + CARS
9	Fat	Healthy connective tissue	SHG + CARS
10	Healthy cells	Healthy connective tissue	SHG + CARS

**Fig. 7:**
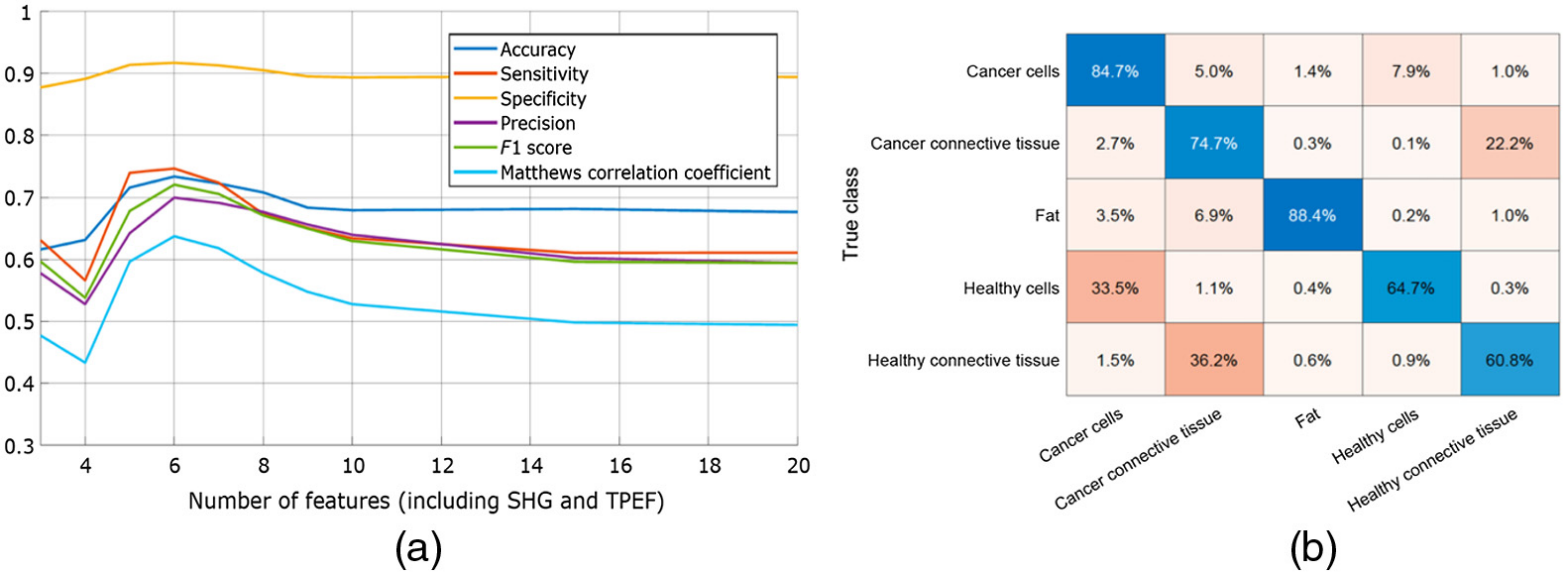
(a) Influence of the number of parameters (including SHG and TPF) on the classification and (b) row-normalized confusion matrix for a data set with six features (SHG, TPF, and CARS 2892, 2943, 3093, and 3192).

A sweep was performed over the number of CARS wavenumbers included in the SVM training set. As shown in Fig. 7, the highest Matthews correlation coefficient (MCC) of 0.64 was achieved with a feature set that contains the SHG signal and four CARS wavenumbers. Increasing the number of features did not result in a better performance instead the performance decreases slightly before stabilizing. The optimal feature set exhibits an accuracy of 0.73; sensitivity of 0.75 and specificity of 0.92.

The types of errors that the algorithm is likely to make can be seen from the confusion matrix in Fig. 7. As expected, the performance on the healthy tissue classes is worse than that of cancer classes, likely due to the small number of healthy samples in the dataset. Even though fat has the lowest number of spectra of any class in the dataset, it still had the highest performance. This is owed to the highly distinguishable CARS spectrum of fat, making it easy to identify even when relatively few examples are available to learn from. Moreover, we can see that the algorithm is most likely to make mistakes between two classes of the same tissue type, i.e., it mistakes cancer cells for healthy cells and cancer connective tissue for healthy connective tissue. This means that good separation is achieved between cells and connective tissue as a whole.

To calculate the predicted majority class per sample, the best performing algorithm (the one with SHG, TPF, and CARS 2892, 2943, 3093, and 3192) was used to make a prediction on unseen data. Afterward, the number of pixels that were identified as cancer (either cancer cells or cancer connective tissue) was summed, as are the pixels that were identified as healthy (either healthy cells or healthy connective tissue). The final sample classification is given by whichever class (i.e., cancer or healthy tissue) has the most pixels. Out of the 16 samples in the dataset, 15 were correctly classified. Only one sample in the dataset was misclassified as cancer tissue, while containing tissue that was classified as healthy by pathology. The feature importance analysis exhibited result similar to the SVM-RFE. The best performance was achieved already when four CARS wavenumbers were combined with the SHG data. The accuracy and sensitivity were 0.87, whereas specificity was as high as 0.95; and MCC, 0.87.

#### 3.3.2 Image reconstruction

**Fig. 8:**
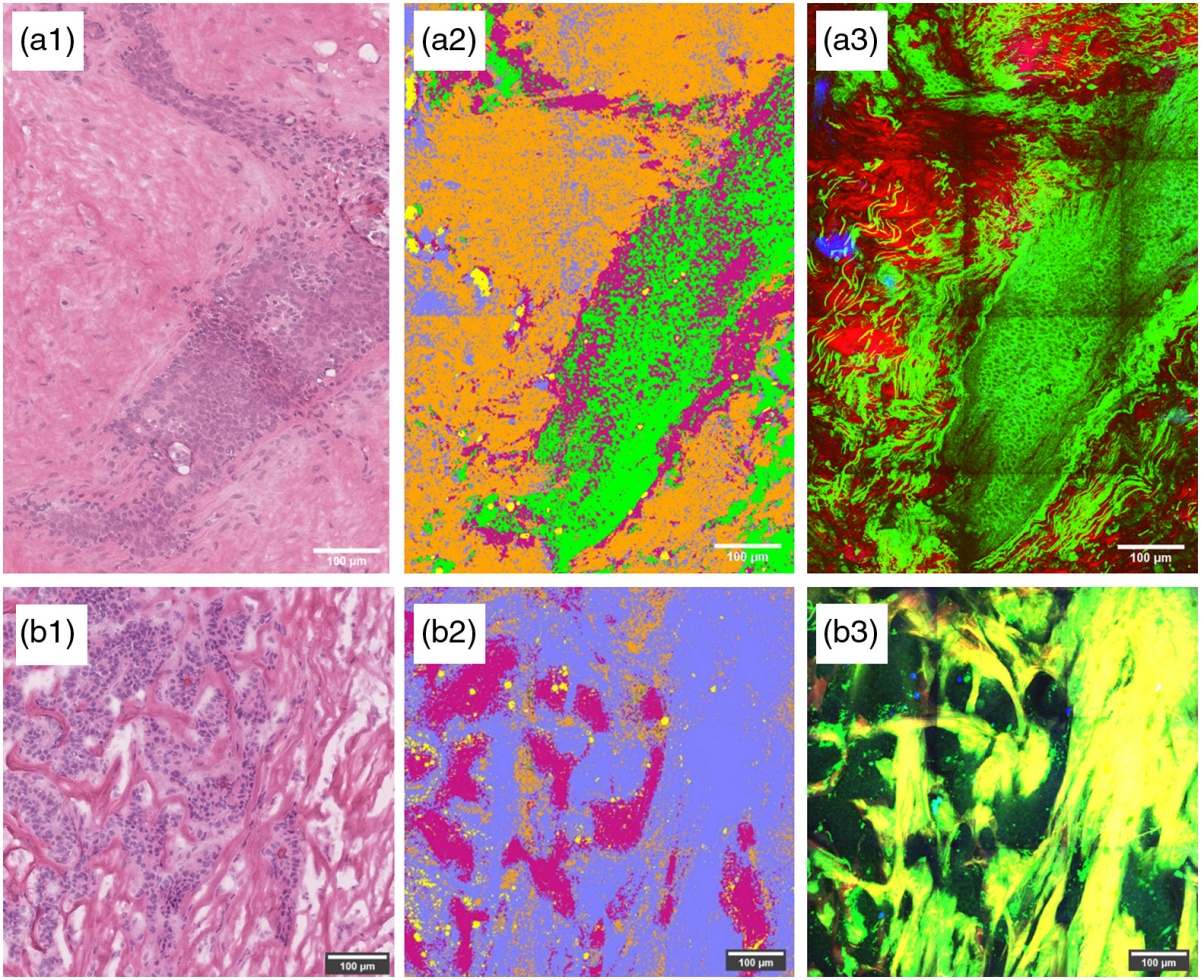
Image classification for (a1)–(a3) a healthy and (b1)–(b3) cancer samples. Column (1) shows the H&E stained counter slice, column (2) shows the color coded predicitions. Orange, healthy connective tissue; green, healthy cells; violet, cancer cells; light blue, cancer connective tissue; and yellow, fat. Column (3) shows an overlay of the SHG, TPF, and CARS image.

To visualize the achieved tissue discrimination results, we applied the trained classifiers to a raw dataset. This visual interpretation of the algorithm’s output can also be compared with H&E annotated slices to check fidelity. As pointed out, the best performance for both machine learning algorithms was achieved with a feature set of SHG, TPF, and four CARS wavenumbers, thus this set was used for classification prior to image reconstruction. The sample data for the reconstructed image were excluded from the training set. Figure 8(a) shows an example of healthy tissue; the connective tissue is generally classified correctly, but the performance on the cells is not as accurate. A lot of pixels were classified as cancer cells. As discussed before, this misclassification can be expected as the algorithm suffers from the shortage of data on normal cells. In the cancer tissue sample [Fig. 8(b)], the cancer cells and cancer connective tissue are classified with good accuracy. Some patches of healthy connective tissue (orange) remains, however, in general the image is conclusive. In both cases, the reconstructed image had clear separation and easily recognizable patterns matching the H&E image.

## 4 Conclusions and Outlook

Using four CARS wavelengths in combination with SHG and TPF directly on excised (thick) tissue, we achieve a sensitivity of 0.87 and a specificity of 0.95 independent of sample treatment. This shows that nonlinear multispectral imaging can be used to accurately determine tumor boundaries. The analysis (using the trained algorithm) only took minutes and could be even faster on a more powerful PC. In BCS, the limiting factor would probably be the acquisition of the four CARS wavelengths, SHG, and TPFE. In our setup, this took 90 min for 5×5  mm of tissue. Using a dedicated system with shorter pulses (1 ps rather than 6 ps) and more sensitive PMTs would likely speed this up by a factor 10. In this work, we did not examine to what extent the resolution could be sacrificed to improve imaging speed over larger areas. Since we do not currently use any spatial information and all relevant areas appear to extend over more than 5 pixels in both directions, the imaging density could be brought down by a factor 10. This would further improve the analysis time by a factor 10. If those two factors are realized, a sample of 10×10  mm could be analyzed in <5  min, which seems acceptable for analysis during BCS. This demonstration brings this technology one step closer to clinical implementation.

## Code, Data, and Materials Availability

The excision materials (pathology slides) are stored following hospital protocol at the Antony van Leeuwenhoek hospital, Amsterdam. The machine learning code and data are available upon request to Prof. Offerhaus.
